# First person – Kai Koike

**DOI:** 10.1242/bio.062784

**Published:** 2026-07-27

## Abstract

First Person is a series of interviews with the first authors of a selection of papers published in Biology Open, helping researchers promote themselves alongside their papers. Kai Koike is first author on ‘ Prey capture strategies relate to prey position in the scale-eating cichlid *Perissodus microlepis*’, published in BiO. Kai conducted the research described in this article while a research assistant in Yuichi Takeuchi's lab at Hokkaido University, Japan. He is now a research assistant in the lab of Matt Smear at the University of Oregon, USA, investigating animal perception and behaviour.

**Figure:**
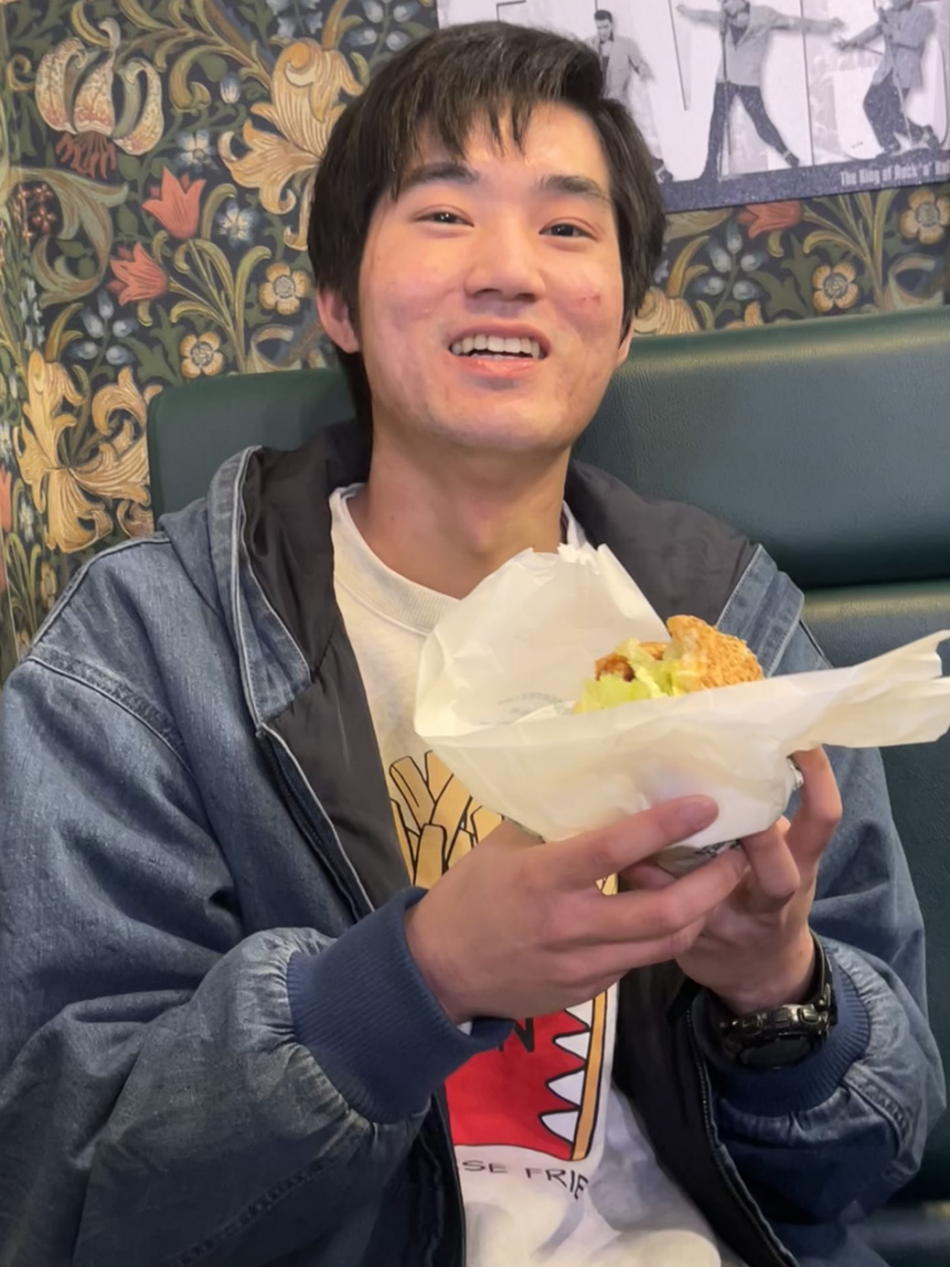
Kai Koike

**Describe your scientific journey and your current research focus**

Ever since I was a child, I have loved animals. I first wanted to become a bear, but the dream never came true. When I was 6 years old, my dream shifted, and I wanted to become a marine biologist. Since then, I have wanted to be a scientist. When I was a first-year college student, I started a behavioural study in fish, which led to my first scientific publication. Upon my continued analysis of fish behaviour via pose-tracking, I began to develop a special interest in the neural mechanisms of behaviour. Building off of this, my current interest lies in neuroscience research where I can study behavioural processes on a microscopic scale and analyse how animals perceive their environment and generate behaviour.

**Who or what inspired you to become a scientist?**

I don't completely know, but I remember watching a TV show that featured a battle between a sperm whale and giant squid. I believe this inspired me a lot for my childhood dream to be a scientist (and animal). My mom also encouraged me to become a scientist, which definitely helped.

**How would you explain the main finding of your paper?**

The scale-eating cichlid has an asymmetrical mouth where the length of the jawbone is longer on one side, which influences the preferred side the fish attacks from. We have determined that this asymmetrical mouth impacts the starting position and direction of the cichlid's attack relative to the position of the prey. We also analysed the movement and trajectory of scale-eating cichlid fish and their prey to uncover the behavioural hunting methods deployed by cichlids. We found that the position of the cichlid relative to the prey led to two main predation strategies: one where the fish attacks from the side and one where it attacks from behind.

**Figure:**
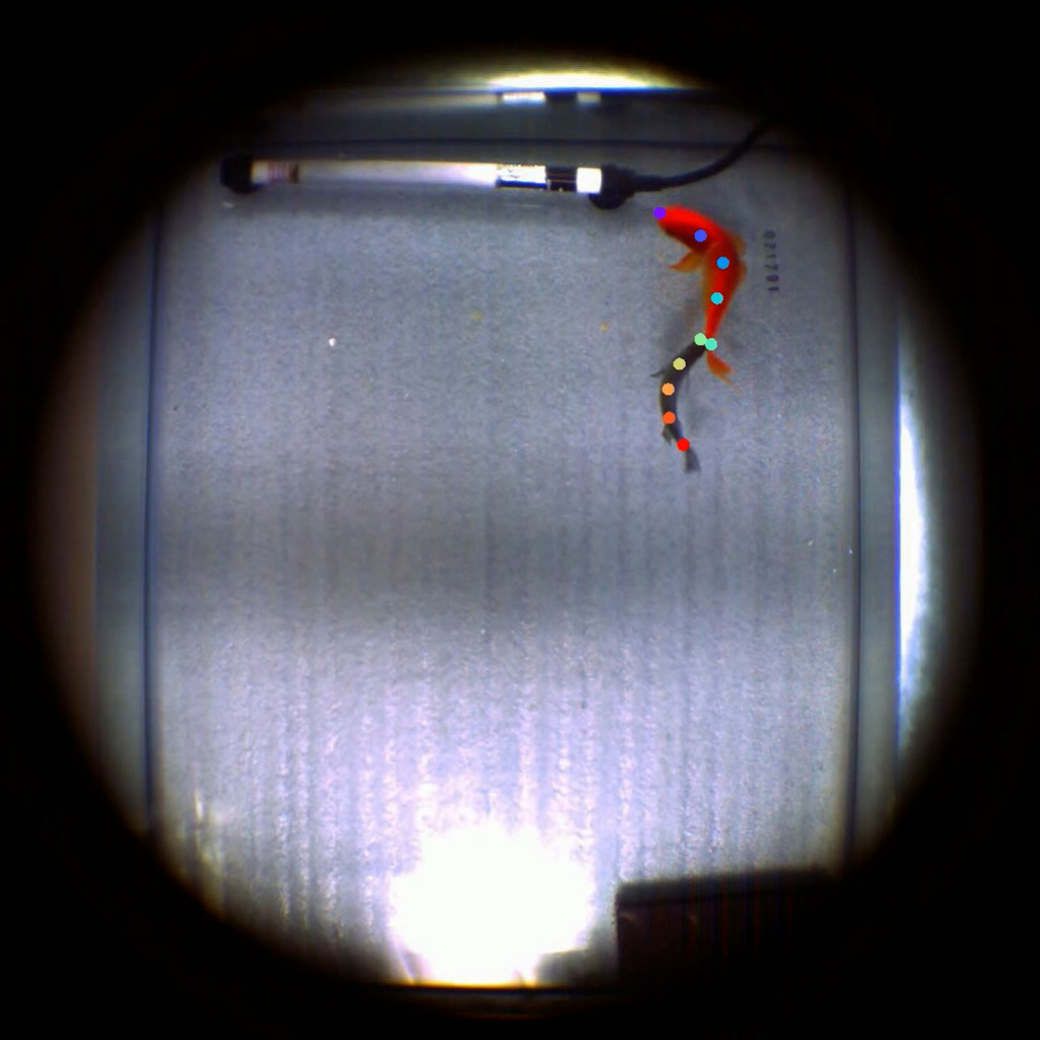
**Cichlid fish with an asymmetrical left-dominant mouth attacks a goldfish from behind, displaying one of the two predation methods identified in the Koike et al. (2026) study.** Five keypoints along the body axis of both the predator and the prey are overlaid on the image.

**What are the potential implications of this finding for your field of research?**

By identifying the two predation strategies of cichlid fish and how they relate to mouth asymmetry, we may begin to think of how left-right dimorphism contributes to the cichlid's evolutionary history and the ecological balance between predator and prey. For example, multiple predation phenotypes could be advantageous since it increases the overall unpredictability of predatory attacks and reduces the prey's ability to effectively predict and escape. Variation in predator behaviour may contribute to maintaining left-right dimorphism by enhancing the advantage of rare behavioural and morphological variants.

**What do you enjoy most about being an early-career researcher?**

At the start of this project, I had little knowledge of fish predation methods. However, as I learned more about the subject and became increasingly familiar with the data, my fascination with the research grew. Each new insight allowed me to see more of the larger picture of the research. Overall, I greatly enjoy the sense of enlightenment that accompanies the scientific process, especially when individual research findings can be connected to broader scientific patterns.

**What's next for you?**

I would like to continue researching predation methods of cichlid fish. I am currently interested in how prey capture behaviour acquires through development from juvenility to adulthood. For my next project, I would like to observe and track changes of preferred predation strategies in juvenile cichlids as they develop into adults in order to see how environmental factors influence the acquisition of specific predation methods.
